# Supplementary material for: Magnetic Data Correction for Fluxgate Magnetometers on a Paramagnetic Unmanned Surface Vehicle: A Comparative Analysis in Marine Surveys
Source: Sensors (Basel). 2025 Jul 21;25(14):4511. doi: 10.3390/s25144511 (PMC12300463; doi:10.3390/s25144511)
Supplement: Supplementary file 1 [file sensors-25-04511-s001.zip › sensors-3710945-supplementary.pdf]

Magnetic Data Correction for Fluxgate Magnetometers on a Paramagnetic USV: A Comparative Analysis in Marine Surveys

Contents of this file

Figures S1 to S8

Table S1

Introduction

The Supporting Information includes additional figures and explanations that complement the key aspects of this study. This allows for a clearer understanding of the research methodology, analysis process, and results, as well as providing access to the data used in the main experiments and additional analysis results.

Contents

Figure S1- Comparison of ambient magnetic field between IGRF-13 model (Alken et al., 2021) and actual measurements at the base station. (Page 2).

Figure S2- shows Frequency and time-frequency analysis (FFT, STFT, CWT) of magnetic data from Sensor 1 and Sensor 2.

Figure S3- CWT analysis of magnetic field measurements taken by a person manually carrying the fluxgate magnetometer in an environment without electromagnetic noise.

Figure S4- Fast Fourier transform (FFT) analysis showing the effects of Butterworth low-pass filtering applied during the anti-aliasing preprocessing.

Figure S5- The corrected magnetic anomaly of the entire track by Equation 3.

Figure S6- Magnetic data correction results for the ferromagnetic R/V Kairei (KR05-17).

Figure S7- Comparison of magnetic anomaly components (X, Y, Z) and yaw change.

Figure S8- Comparison of corrected shipborne fluxgate magnetometer data with towed scalar magnetometer data over a cross-over track line.

Table S1- Comparison of correction coefficients using different suggested methods for the ferromagnetic R/V Kairei.

Comparison between Base Station Data and the IGRF-13 Model

We confirmed that the data collected from the base station magnetometer (geomagnetic observatory) during the survey period exhibited only typical diurnal variations, and no short-wavelength geomagnetic disturbances related to solar flares were observed. The differences between the measured values and the IGRF model were 0.35% for the x-component, 1.84% for the y-component, and 0.25% for the z-component. Therefore, we can conclude that there were no issues with using the IGRF model for correction in this study. The differences between the measured geomagnetic field and the IGRF model were within a small range, indicating that the IGRF model is suitable for the correction in this study.

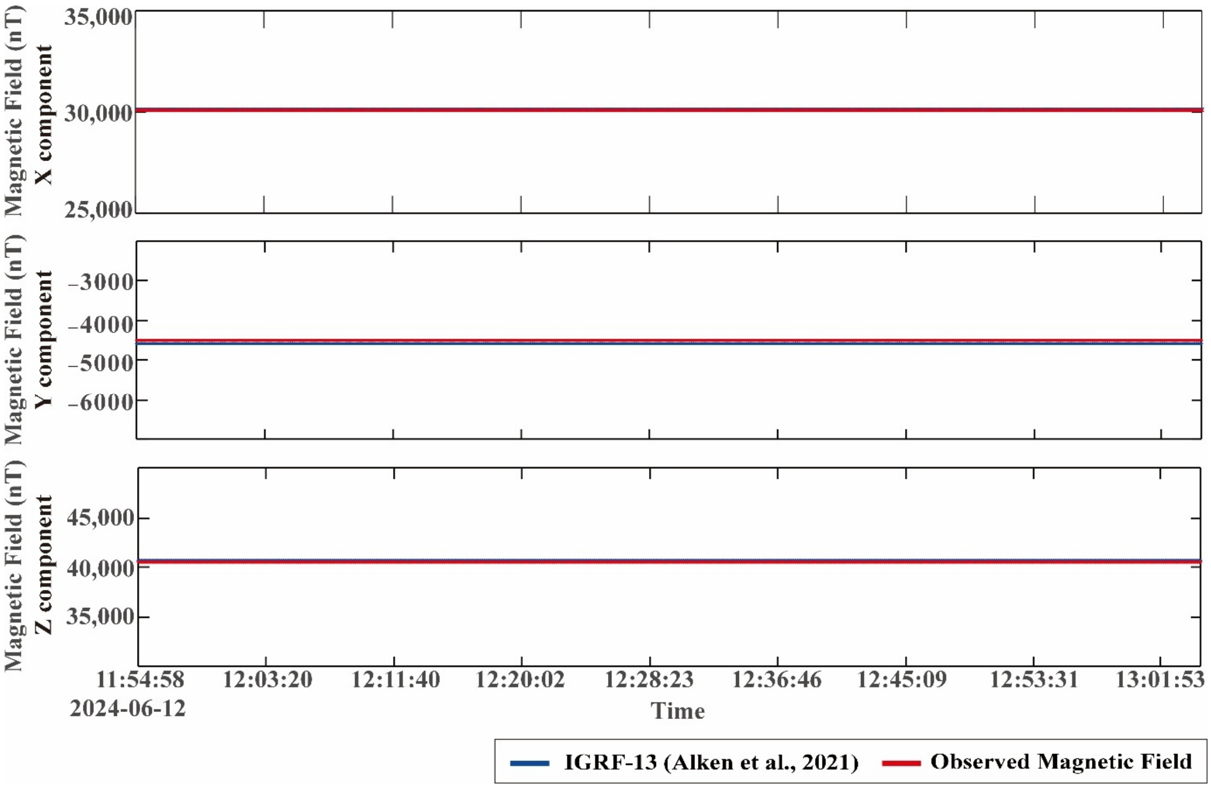

Figure S1. Comparison of ambient magnetic field between IGRF-13 model (Alken et al., 2021) and base station at the same location.

Sensor Characteristics and Comparisons

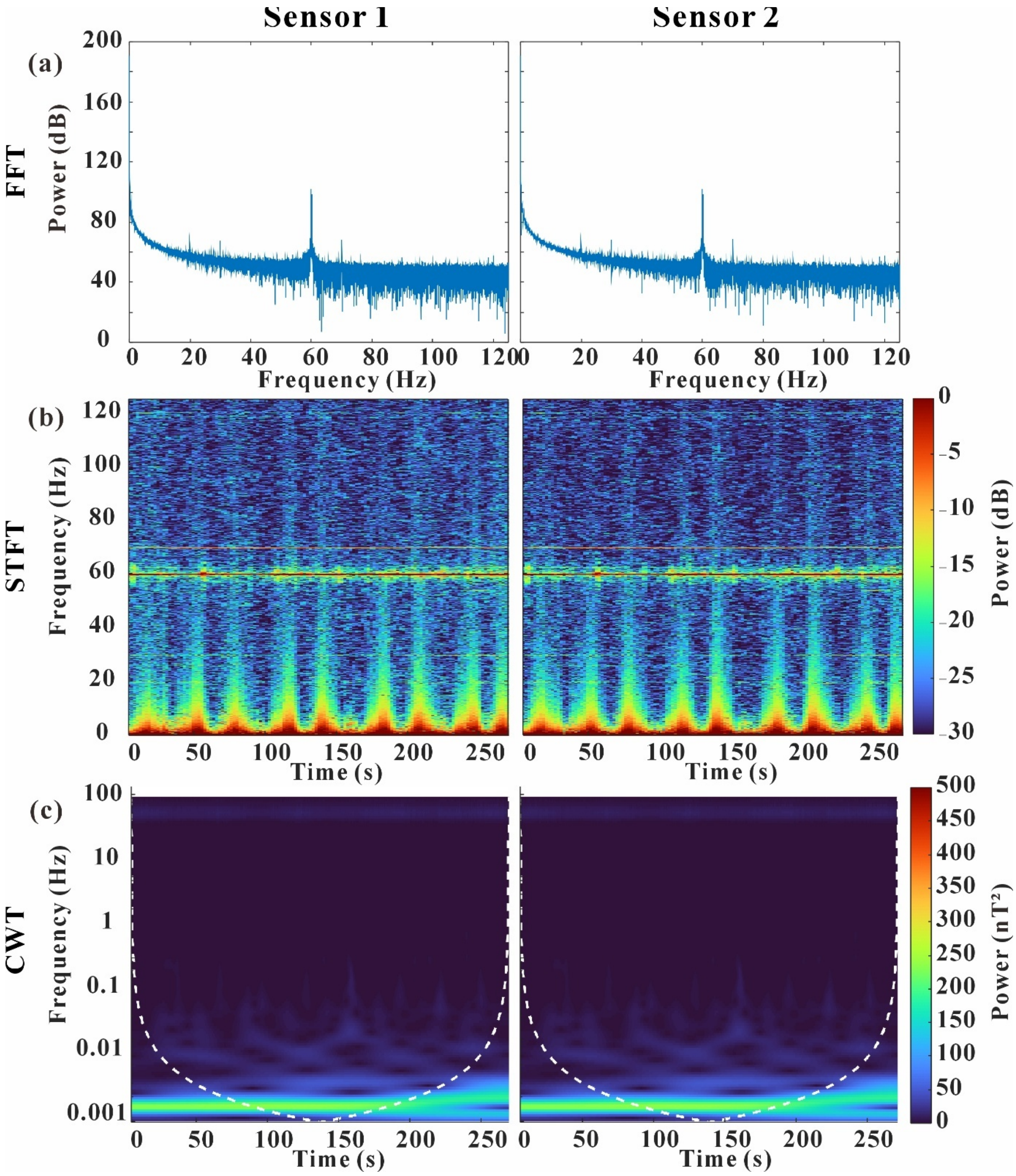

Figure S2. Frequency and time-frequency analysis (FFT, STFT, CWT) of magnetic data from Sensor 1 and Sensor 2, acquired during a land-based survey on a beach near the USV survey area. (a) Fast Fourier Transform (FFT) spectrum in the frequency domain, showing the amplitude distribution across frequencies up to 125 Hz. The y-axis represents the amplitude

on a logarithmic scale. (b) Short-Time Fourier Transform (STFT) spectrogram, illustrating the time-frequency distribution of the magnetic signal during the survey period. The color scale indicates signal intensity in decibels (dB), with red representing higher intensities and blue indicating lower intensities. (c) Continuous Wavelet Transform (CWT) in the time-frequency domain, displaying the time-frequency distribution of the magnetic signal during the survey period. The color scale represents signal intensity in (nT^2) units, with red indicating stronger signals and blue representing weaker signals. The white dashed line in the CWT image represents the confidence interval of the analysis.

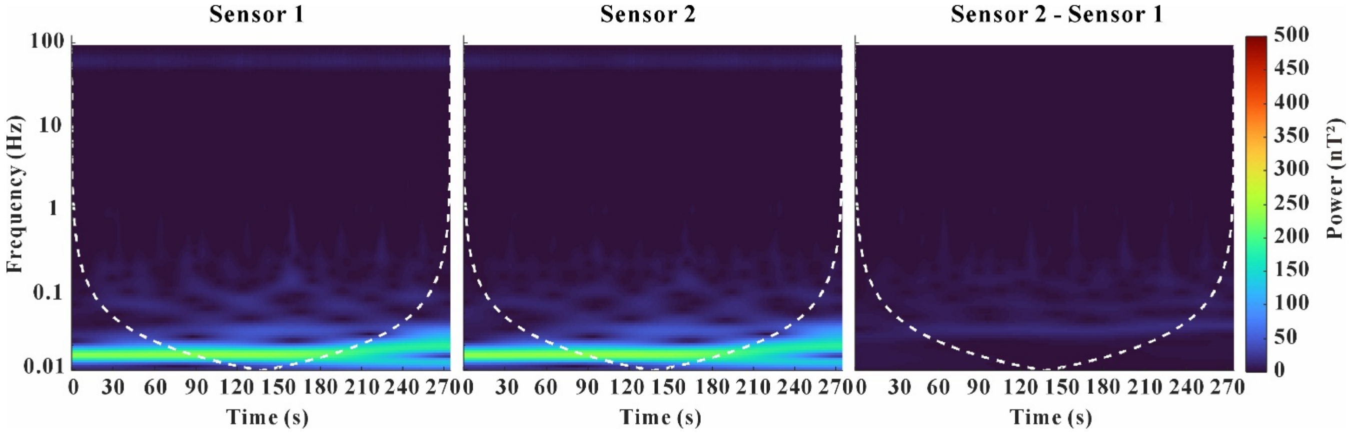

Figure S3. CWT analysis of magnetic field measurements taken by a person manually carrying the fluxgate magnetometer in an environment without electromagnetic noise. The color bar is displayed with a linear scale, and the unit is nT^2, with red representing strong components and blue representing weak components. The white dashed line indicates the confidence interval.

Data Integration and Anti-Aliasing

A necessary step during data preprocessing involved the integration of magnetometer data, originally sampled at 250 Hz, with the ship's motion components (roll, pitch, yaw), recorded at 50 Hz, and GPS data, captured at 10 Hz. To achieve this integration, the magnetometer data required downsampling to a consistent 10 Hz rate.

We recognized that this downsampling process could introduce signal distortion through aliasing, a phenomenon where signal frequencies higher than the Nyquist frequency (5 Hz for a 10 Hz sampling rate) are erroneously represented as lower frequencies. To prevent such

distortion, we implemented a low-pass filter prior to downsampling, setting the cutoff frequency at 4.5 Hz, slightly below the critical 5 Hz Nyquist frequency.

To evaluate the efficacy of this anti-aliasing filter, we performed a Fast Fourier Transform (FFT) analysis on the filtered data. Figure S4 presents the results of this FFT analysis. These results confirm a significant attenuation of frequency components above the 5 Hz Nyquist threshold, while the power spectral density within the lower-frequency bands remains consistent with the original signal. This analysis indicates that the applied low-pass filter effectively removed the undesirable high-frequency components, thereby preserving the integrity of the signal intended for further analysis.

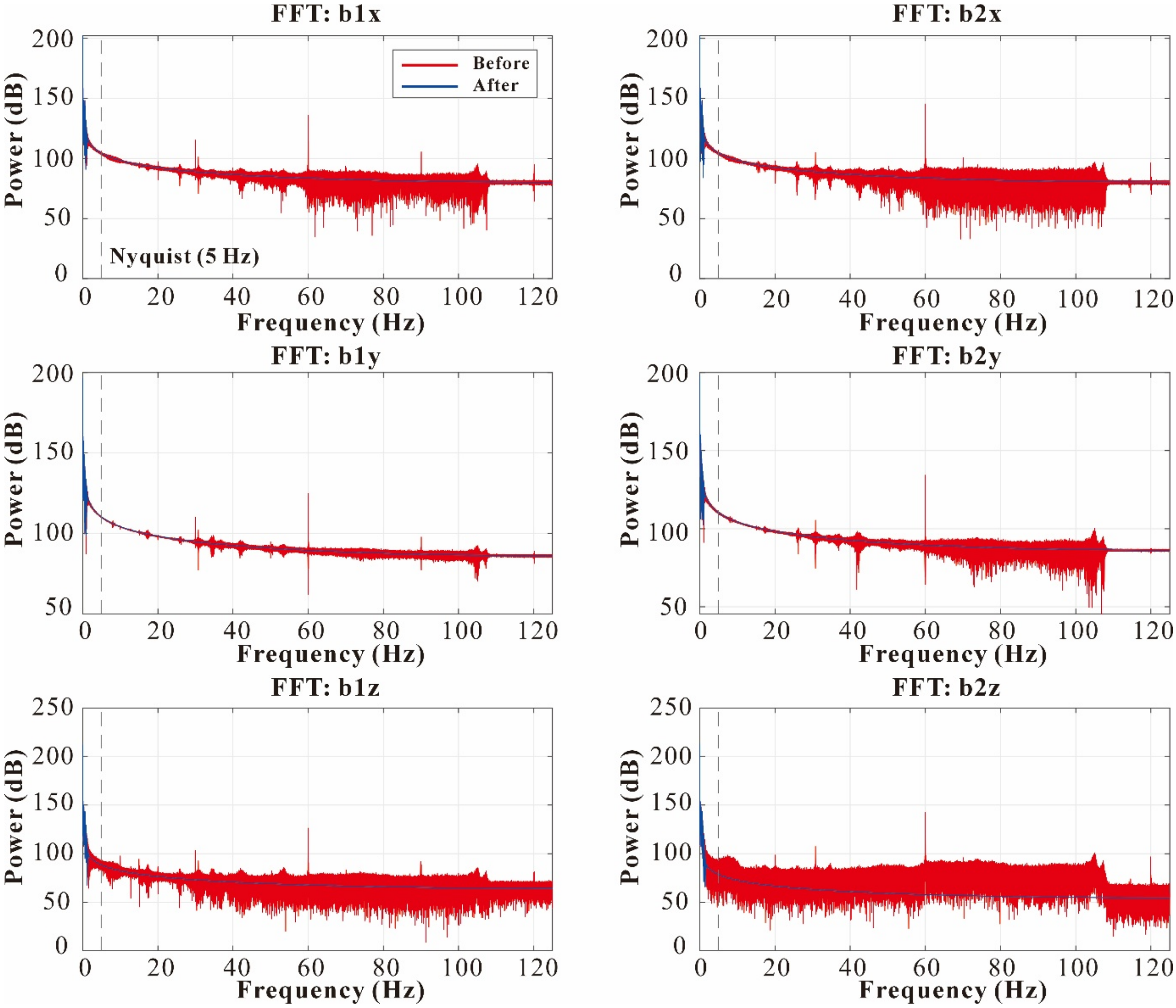

Figure S4. Fast Fourier transform (FFT) analysis showing the effects of Butterworth low-pass filtering applied during the anti-aliasing preprocessing. The frequency domain representations demonstrate the removal of high-frequency components in both sensors when compared to unfiltered data (blue lines: before filtering; red lines: after filtering). The panels display the three orthogonal components (x, y, z) for Sensor 1 (b1x, b1y, b1z) and Sensor 2 (b2x, b2y, b2z), with the Nyquist frequency (5 Hz) indicated by the vertical dashed line.

Geomagnetic Field Correction and Anomaly computation

Figure S5 shows the corrected sum of the geomagnetic field across the entire track, applying Equation 3 from Seama (1992). The vector components of the geomagnetic field exhibiting rapid changes are observed to remain nearly constant after the correction. Subsequent to this initial correction step, we computed the geomagnetic anomaly by subtracting the International

Geomagnetic Reference Field (IGRF) model.

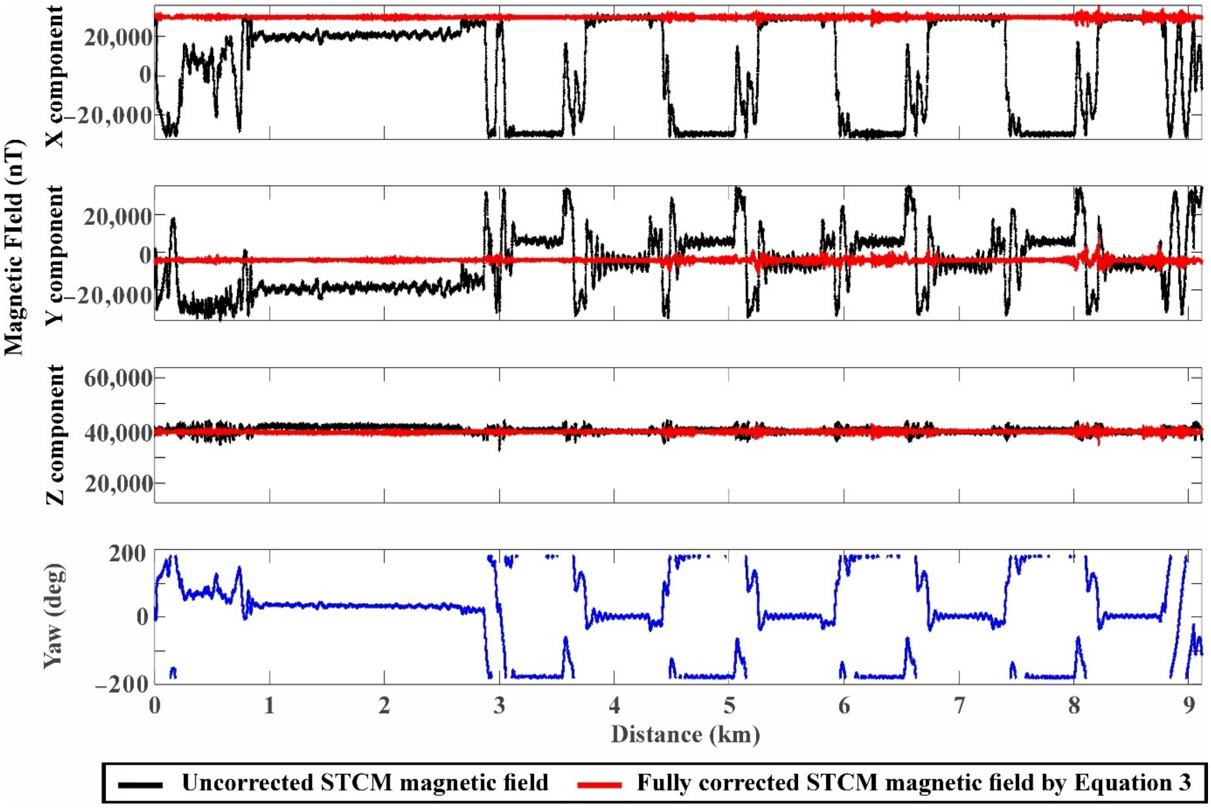

Figure S5. The corrected magnetic anomaly of the entire track by Equation 3. The black solid line represents the original data, the red solid line represents the corrected data, and the blue solid line represents the yaw degree.

Attitude and Magnetic Correction for ferromagnetic Research Vessel

Table S1. Comparison of correction coefficients using different suggested methods for the ferromagnetic R/V Kairei.

**Correction coefficients from Equation 2**
$A = \begin{bmatrix} 0.9063 & -0.0656 & -0.1731 \\ 0.0655 & 0.7641 & 0.0100 \\ 0.0277 & -0.0048 & 0.9677 \end{bmatrix} \quad \mathrm{H_p} = \begin{bmatrix} -148.31 \\ 6292.28 \\ 9757.72 \end{bmatrix}$
**Correction coefficients from Equation 3**
$B = \begin{bmatrix} 1.1024 & 0.0933 & -0.1913 \\ -0.0927 & 1.3003 & -0.0343 \\ -0.0310 & 0.0040 & 1.0106 \end{bmatrix} \quad \mathrm{H_{pb}} = \begin{bmatrix} 1346.43 \\ -7329.22 \\ -9490.24 \end{bmatrix}$
**Correction coefficients from Equation 4**
$S = \begin{bmatrix} 1.1698 & 0 & 0 \\ 0 & 0.3455 & 0 \\ 0 & 0 & 1.0831 \end{bmatrix}$

$$P = \begin{bmatrix} 1 & 0 & 0 \\ -0.1338 & -0.9910 & 0 \\ 0.9048 & -0.2079 & 0.3716 \end{bmatrix} \quad O = \begin{bmatrix} -330 \\ 2742 \\ -651 \end{bmatrix}$$

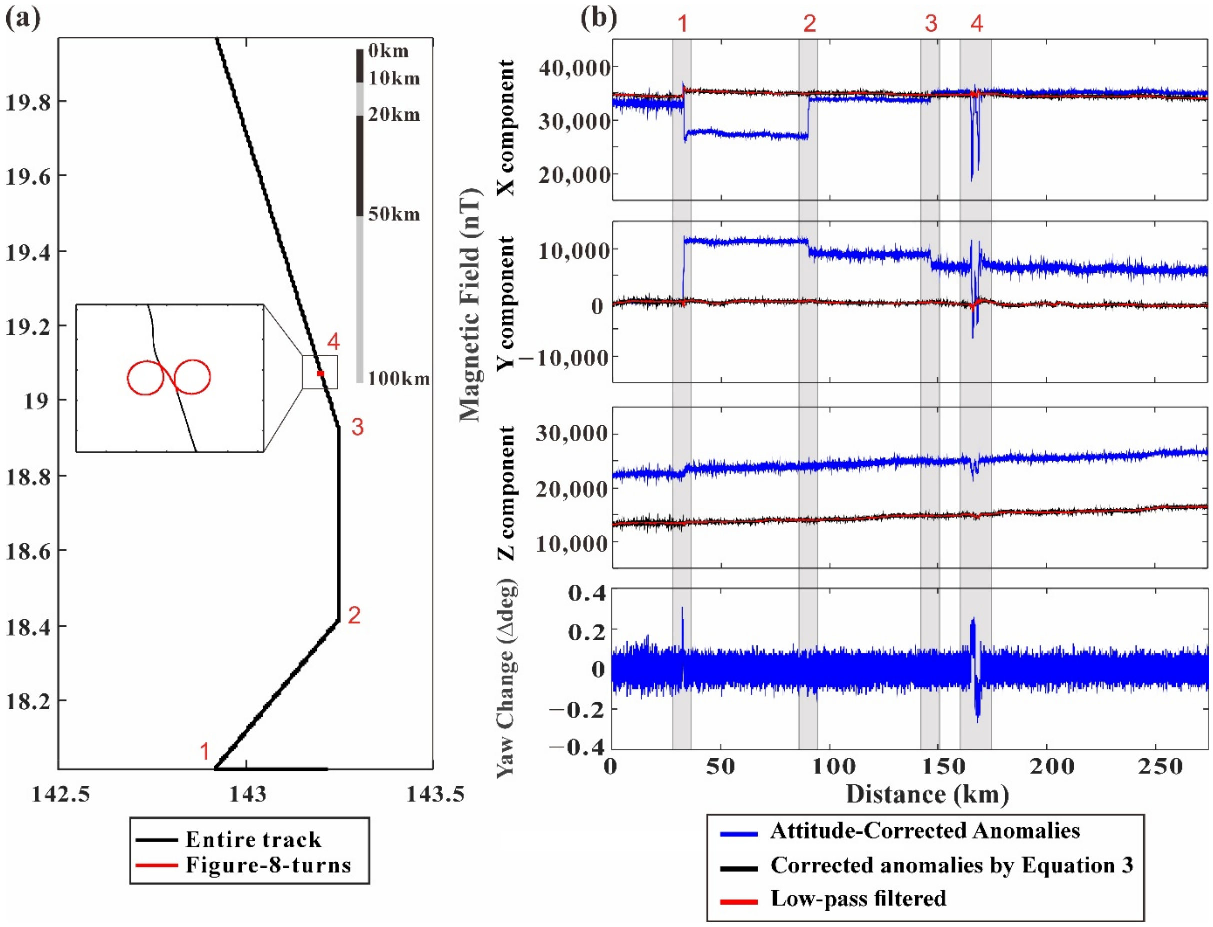

Figure S6. Magnetic data correction results for the ferromagnetic R/V Kairei (KR05-17). These results were reconstructed using data from Seama (2005). (a) the cruise survey track with inset showing figure-eight correction maneuvers (red). Red numbers indicate the locations where ship's azimuth changed. (b) Magnetic field components (X, Y, Z) and Yaw change plotted against distance along the track. Lines show attitude-corrected anomalies (blue), anomalies corrected using Equation 3 (black), and low-pass filtered corrected anomalies (red). Note the significant difference between attitude-corrected and ship's magnetization-corrected data by Equation 3, reflecting the strong magnetic influence of the ferromagnetic hull.

Comparison of magnetic signal characteristics between a paramagnetic USV and a ferromagnetic Research vessel

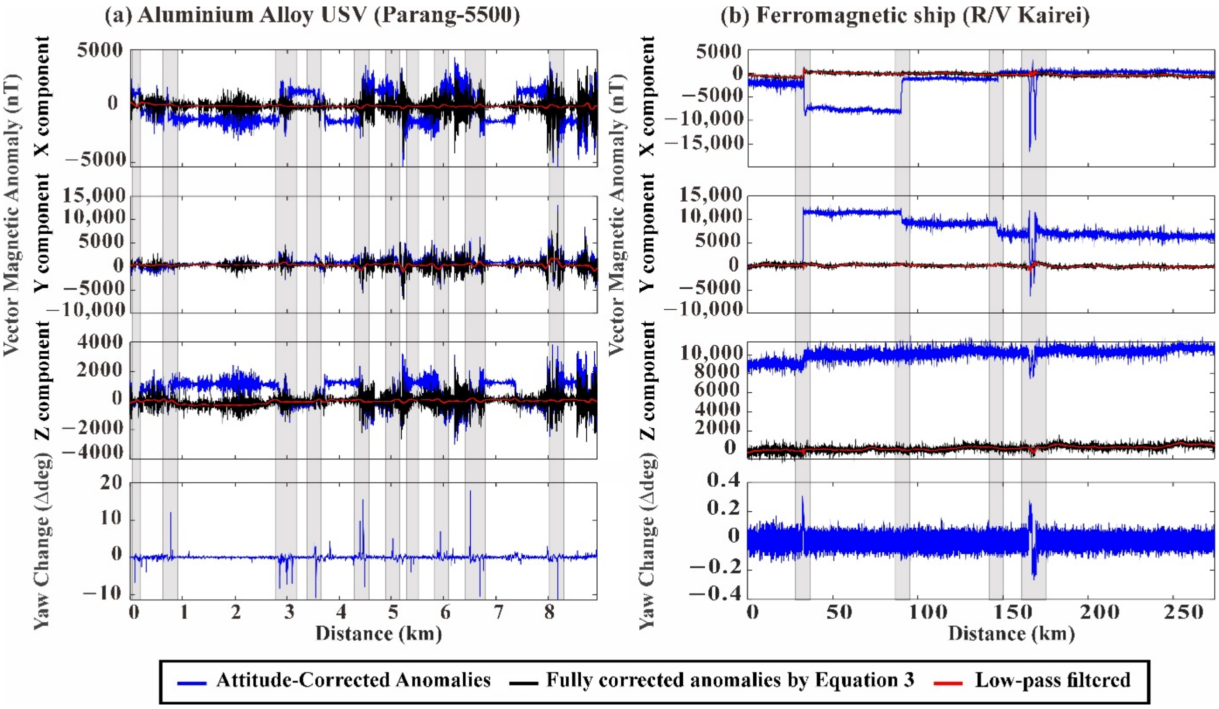

Figure S7. Comparison of magnetic anomaly components (X, Y, Z) and yaw change from (a) an aluminum-alloy USV (Parang-5500) and (b) a ferromagnetic research vessel (R/V Kairei), highlighting the impact of full correction (Equation 3) versus attitude correction alone. Blue: attitude-corrected; black: fully corrected; red: low-pass filtered. Gray shading: significant yaw change. Note differing scales reflecting different survey environments (USV: shallow coastal water; R/V Kairei: open ocean).

Verification of Correction Accuracy and Reliability

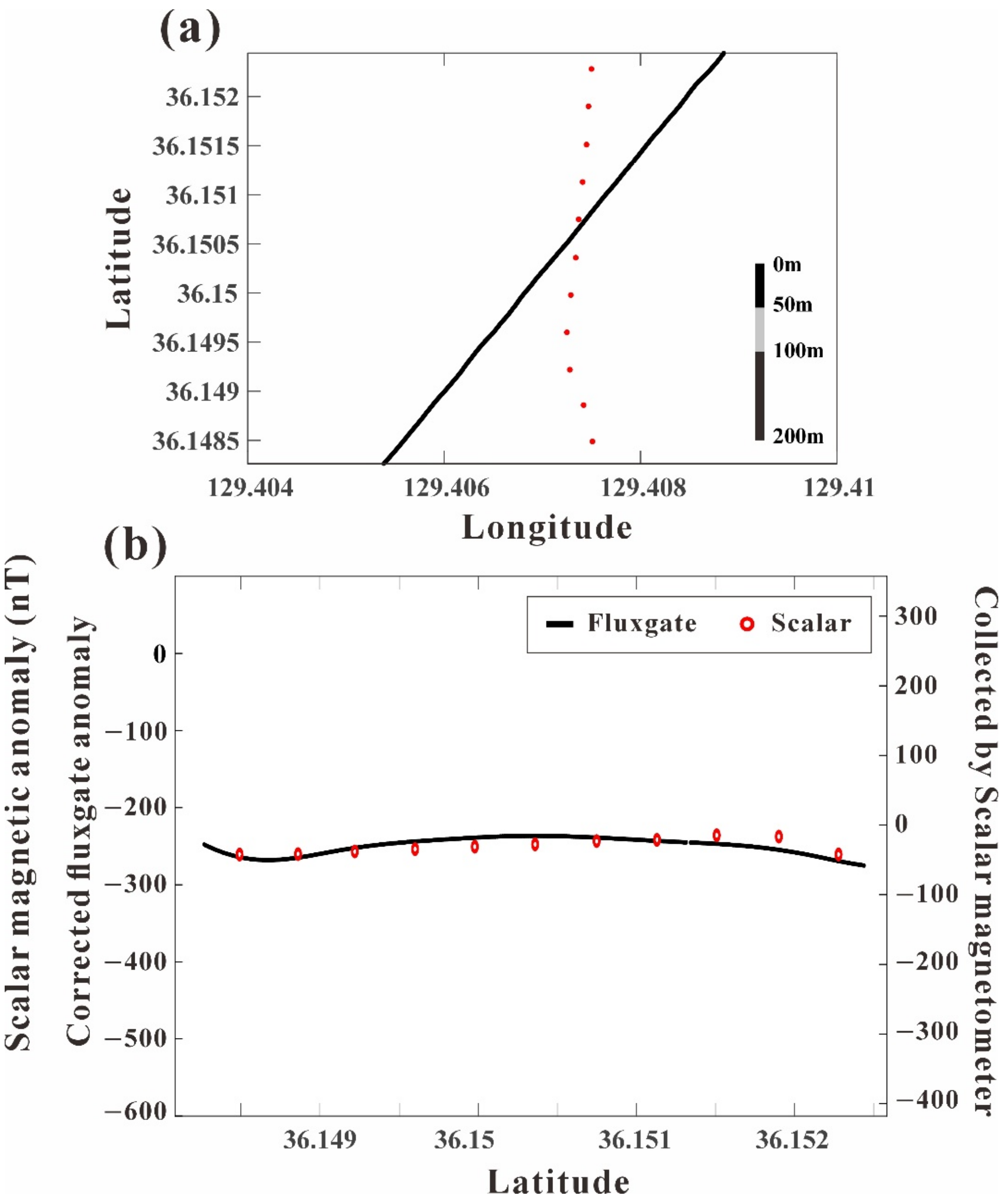

Figure S8. Comparison of corrected shipborne fluxgate magnetometer data with towed scalar magnetometer data over a cross-over track line. (a) displaying cross-over tracklines between the USV survey trackline (black solid line) and the location of crossing measurements acquired by the towed scalar magnetometer (red dots). (b) comparison of geomagnetic anomaly after correction. The black solid line show the anomaly derived from the corrected USV fluxgate vector data (left axis), while the red circles represent direct measurements after daily correction from the towed scalar magnetometer (right axis). Note the good agreement in the trend, but an approximate 200 nT offset in absolute values between the two systems.

References

1. Seama, N. Studies of Vector Geomagnetic Anomalies in the Oceans. *J. Jpn. Soc. Mar. Surv. Technol.* **1992**, *4*, 41–49. https://doi.org/10.11306/jsmst.4.2_41.
2. Seama, N. *Cruise Report of KR 05-17*; JAMSTEC: Yokosuka-city, Japan, 2005. https://doi.org/10.17596/0001047.
